# Myocontrol in Aging

**DOI:** 10.1371/journal.pone.0001219

**Published:** 2007-11-21

**Authors:** Eric J. Fimbel, Martin Arguin

**Affiliations:** 1 Fatronik Foundation Research Center, Laboratoire Cognition et Facteurs humains EA 497, Institut de Cognitique, Bordeaux, France; 2 Institut Universitaire de Gériatrie de Montréal, Montréal, Canada; 3 Department of Psychology, Université de Montréal, Montréal, Canada; Ordway Research Institute, United States of America

## Abstract

Myoelectric (EMG) signals are used in assistive technology for prostheses, computer and domestic control. An experimental study previously conducted with young participants was replicated with elderly persons in order to assess the effect of age on the ability to control myoelectric amplitude (or myocontrol). Participants performed pointing tasks as the myoelectric amplitude was captured by a surface electrode in two modalities (sustained: stabilize the amplitude after reaching the desired level; impulsion: return immediately to resting amplitude). There was a significant decrease of performance with Age. However, the patterns of performance of young and aged were noticeably similar. The Impulsion modality was difficult (high rates of failure) and the speed-accuracy trade-offs predicted by Fitts' law were absent (bow-shaped patterns as function of target amplitude instead of logarithmic increase). Conversely, the reach phase of the Sustained modality followed the predictions of Fitts' law. However, the slope of the regression line with Fitts' index of difficulty was quite steeper in aged than in young participants. These findings suggest that 1) all participants, young and aged, adapt their reaching strategies to the anticipated state (sustained amplitude or not) and/or to the difficulty of the task, 2) myocontrol in aged persons is more fragile, i.e., performance is markedly degraded as the difficulty of the task increases. However, when individual performance was examined, some aged individuals were found to perform as well as the young participants, congruently with the literature on good aging.

## Introduction

The present study investigates how age affects the ability to control myoelectric signals, or *myocontrol*. Myoelectric signals (EMG) are used for controlling a variety of devices [1], like prostheses [2], domestic systems [3], computer interfaces, e.g., [4], [5], or mobile devices like cell phones, e.g., [6]. Myoelectric signals are of special interest for disabled users because they can be captured from any preserved body part, e.g., forehead in case of quadriplegia, controlateral shoulder in case of arm prostheses.

The ability to control myoelectric signals (myocontrol) is of importance for the usability of the foregoing devices. This capacity determines the rate of command entry, the proportion of errors and the precision of the commands. Temporal precision is required in scanning interfaces in which the user sends a command when the desired object is highlighted [7]. Amplitude precision is required when different levels of amplitude correspond to different commands, such as *k-switc*h interfaces in which the analog amplitude is converted into discrete commands by means of multiple thresholds [7].

Like in movement, there are trade-offs in myocontrol, between the speed and the accuracy or the precision demand [8]. However, the nervous system evolved to control the geometry, kinematics and dynamics of the body and the limbs, whereas myolectric signals are only a collateral outcome of motor activity. As a result, myocontrol is by no means natural: myoelectric signals and the devices that convert them into commands can be considered *artificial effectors*. Myocontrol may thus require specific strategies that have never been practiced before, even for the most basic actions, namely reaching a determined level of myolectric amplitude or sustaining a steady myoelectric amplitude.

A study conducted with young healthy participants [9] documented differences between myo- and motor control. Participants had to reach some determined myoelectric amplitude under visual guidance by means of a fast muscular contraction. In a first condition, participants released the contraction immediately (impulsion modality); in a second condition, participants sustained the contraction so that the amplitude was steady for at least one second (sustained modality). The slow execution times observed in both conditions and the high rate of error in the impulsion modality confirmed that myocontrol is difficult (even with the reasonable tolerances used in the study, i.e., 25% or 12.5%).

When performance in the impulsion modality (execution time, rate of errors) was examined as a function of myoelectric amplitude, a bow-shaped pattern was observed, in clear contrast to the logarithmic increase predicted by Fitts' law [10]. This result was unexpected, because Fitts' law has been reasonably well verified with similar feedback conditions (vertical monitor), artificial effectors like a mouse or a joystick [11], and isometric conditions [12]. The fact that performance could not be described by means of a simple function of amplitude suggests that participants used specific “strategies” (or synergies) adapted to target amplitude and to the anticipated final state (maintain the contraction or relax immediately).

However, the foregoing results were obtained with young healthy participants whereas the users of adaptive technologies are presumably older than the remainder of the population. A minimal justification of this statement is based on probabilities: i) the cumulative probability of accident or disease that may cause disabilities increases across the lifespan, and ii) disabilities that require adaptive technologies are generally not reversible. The effects of age on cognition have been repetitively documented but whether they result from a general decline in mental operations [13] and/or from perceptual deterioration [14] is still debated. The effects of age on memory [15], attention [16] and motor skills [17] have also been documented. However the way in which age specifically affects myocontrol remains an open issue.

A variety of factors may contribute to an age-related decline in myocontrol. Aging is associated with a series of changes in the peripheral nervous system and the motor units: loss of spinal motoneurons, decrease in the conduction velocity of axons, loss of muscle fibers, increase in the size of motor units and reorganization of motor units [18], [19]. With fewer and coarser motor units, the myoelectric signals are likely to change in older persons. This was confirmed by several studies [20], [21], [22] Also, age-induced changes of central motor control possibly affect myo-control.

An aspect of importance is the control of timing and synergies. The amplitude of the myoelectric signal captured by a surface electrode depends on intra- and extra- muscular synergies (e.g., co-contractions), and, at a finer level, on the patterns of discharge and the synchronicity of the motor units [23], [24]. Controlling the synergies of multiple sources of signal indeed provides many degrees of freedom, i.e., flexibility in the control of myoelectric amplitude. However, synergies themselves may be difficult to control. In motor control, a decrease of finger synergies with age was reported in isometric tasks, force production [25] and grasping [26]. Darling & Cooke [27] examined EMG activity during pointing tasks and found that the increase of trajectory variability with age was accompanied by increased co-contraction and incorrect timing of the myolectric activity of the antagonistic muscle (see [28], [29] for details on the myoelectric activity during rapid movements). A decline in the capacity of controlling synergies and timing may expectedly decrease the capacity of myocontrol of aged persons.

However, it has been reported that in motor tasks, aged persons seem to employ adaptive strategies that counterbalance (at least partially) their reduced capacities [30]. Indirect evidence of age-dependent strategies in motor tasks is provided by functional imaging. There exist age-related differences in the patterns of cortical activity during motor tasks, but whether these differences correspond to deliberate age-dependent strategies and/or to age-dependent neuronal reorganization is still unclear [31]. In the specific context of pointing tasks, additional evidence of age-dependent strategies is provided by speed-accuracy trade-offs. For instance, [32] found different trade-offs in young and aged participants in a 2D pointing task. To be more specific, target size and target amplitude affected differently the initial phase (transport) and the final phase (landing) in young and aged participants. In addition, the repetitiveness of the tasks realized in experimental conditions may also elicit adaptive strategies in elderly participants, in order to compensate fatigue, as reported by [33].

The present study was conducted in order to document the effects of age on myocontrol. Aged participants performed the same tasks as the young participants in [9]. The effects of Age on Performance, and the interactions between Age, Precision demand and Amplitude were examined, as well as the eventual differences in speed-accuracy tradeoffs between young and aged participants.

## Materials and Methods

The present study is the second part of an experiment which was conducted with young participants [9]. In the first part of the experiment, the tasks were performed by two groups of participants, each group using a different electrode placement: forehead (above the eyebrow) or hand (thenar eminence). In the second part, presented here, the same tasks were executed by aged participants, but we limited ourselves to the hand placement (the forehead placement was abandoned because it was uncomfortable for several aged participants). These hand data from elderly participants were contrasted to those previously obtained in young participants in order to assess the effects of age on myocontrol.

### Participants and apparatus

Participants were 23 healthy elderly volunteers (age = 71.7 years, σ = 5.3, 9 males, 1 left-handed) and 19 young volunteers (age = 24.3 years, σ = 3.9, 13 males, 2 left-handed). None of the participants had a history of motor, neurological or perceptual deficits. Twenty young participants initially executed the test, but the data of one of them was not exploitable for technical reasons. For the aged participants, we tested more than our initial target of 20 to compensate for possible data loss, but all data were complete. All participants gave written informed consent prior to the experiment. The protocol (for young and aged) was previously approved by the Ethic Committee of Institut universitaire de gériatrie de Montreal (ref. 2003-0302).

#### Electrode montage

**Figure 1:**
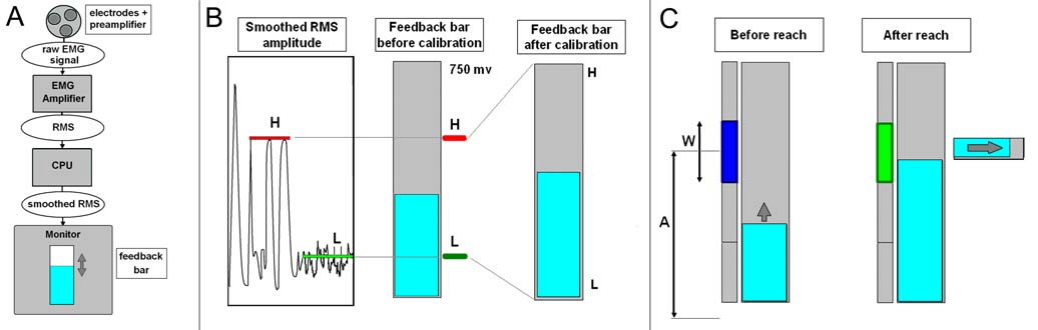
Experimental setup. *a)* Signal capture. Raw EMG signal filtered in the band 25–450 Hz, converted in RMS, sampled at 1 khz and smoothed by averaging in bins of 50 ms. Feedback bar: width 2 cm, maximal height 20 cm, distance from the eyes 60±10 cm, refresh rate 20Hz. *b)* Feedback calibration. Before calibration, the bar represents the voltage of the smoothed RMS signal in the range 0–750 ms. After calibration, the bar represents the signal normalized in the range *L, H*. *c)* Pointing task. Feedback bar represented before- and after the reach in the sustained modality. *A, W*: amplitude and width of the target. For normalization, we considered that *L* was at distance W/2 from the rest level, i.e., for target k, *A = W/2+(k−1)W+W/2 = kW*. Horizontal progress bar: time elapsed in the target.

The montage (Fig. 1a) consisted of three dry silver electrodes spaced 2 cm apart (two differential electrodes and one common electrode) and a preamplifier (Neurodyne AE-104). The montage was placed on the palm of the dominant hand, on the thenar eminence and maintained by an elastic band. The myoelectric amplitude was mainly controlled by means of thumb extensions and/or isometric pressure of the thumb and the forefinger (the electrodes captured activity from abductor pollicis brevis, flexor pollicis brevis, opponens pollicis, adductor pollicis transversalis, and marginally, from other hand muscles, such as the first and second lumbricali and opponens quinti digiti).

The preamplifier amplified and filtered the signal in the band 25–450Hz, then an amplifier (Neurodyne system/3) converted the signal into RMS (Root Mean Square), in the range 0–750 mV. A computer captured the signal at a frequency of 1 kHz. The EMG signal captured in this way is highly variable and difficult to control, it was thus smoothed (averaged over bins of 50ms) and displayed on the computer monitor with a refresh rate of 20Hz. The signal processing introduced a delay (lag) of 55ms (σ = 8ms) between the emission of the EMG signal and the display on the monitor. This lag is acceptable according to the literature on pointing tasks, e.g., [34].

### Training and Calibration

As soon as the electrode montage was placed, the participant could visualize the EMG amplitude as a vertical bar on the computer screen. He or she was instructed to “find a way to control the feedback bar by contracting his or her muscles”. No specific instructions were given regarding which muscles to contract. The training ended by common agreement, i.e., the participant and the experimenter considered that minimal control was obtained. Participants could also retrain during the rest period between blocks of trials. The average duration of the first training phase was 226 s (σ = 93 s) for young participants and 318 s (σ = 219 s) for aged participants.

After the training phase, the gain (i.e., the ratio of the height of the feedback bar vs. the voltage of the myoelectric signal) was calibrated so that the range of amplitudes that the participant could effectively produce corresponded to a normalized spatial extent on the monitor (Fig. 1b). By doing so, the visual feedback was the same for all participants whatever the differences due to the strength, the muscular mass, the conduction of the skin and/or the variations of electrode placement. The gain was adjusted in two steps. First, an upper bound *H* of the EMG amplitude was determined. The Participant produced a contraction at maximal amplitude, which (s)he was generally unable to reproduce in consecutive attempts. The process was repeated with weaker contractions until the participant was able to produce the same amplitude on three consecutive trials. This grants that the participant is capable of producing levels above *H* for short periods of time.

Then, a lower bound *L* of the EMG amplitude was determined by instructing the participant to relax so that the amplitude was minimal (the visual feedback was of considerable help for this task). *L* was then determined as the average rest amplitude plus one standard deviation. Note that it is possible to produce levels below the level of variability at rest for short periods of time (as it occurs with EEG, when signals are below the level of noise). By calibrating in this way, the range [*L, H*] coarsely represents the amplitudes that can be effectively sustained, while still allowing the user to overshoot *H* or undershoot *L.*

### Task

Participants had to reach a “target” of a determined “diameter”, placed at a variable “distance” that determined the “amplitude of the movement” (Fig. 1c). The “target” was a vertical interval on the monitor; and “reaching the target” consisted in placing the feedback bar into this interval. Note that the concepts of the task, “target”, “distance”, “diameter”, “movement”, etc. can be expressed either in terms of EMG amplitudes or in terms of spatial extents on the monitor. Furthermore, we can eliminate specific units (centimeters, volts) by considering proportions instead of distances. For instance, the “total range” [0, 1] represents the maximal length of the feedback bar as well as the range of effective EMG amplitudes [*L, H*].

There were two conditions of precision demand. In the *low precision* condition, there were 4 targets, i.e., the total range was divided into 4 intervals of diameter 25% each. For simplicity, the distances were determined from a “virtual target” situated just below the total range. With this convention the distances were 25%, 50%, 75% and 100% of the total range, and Fitts' indexes of difficulty Id = log_2_(2 distance/diameter) became log_2_(2), log_2_(4), log_2_(6) and log_2_(8) respectively. The reference point chosen here corresponds to an ad-hoc formulation of Fitts' index of difficulty *Id*. This is not a problem in the present case, because we only look for linear correlations between *Id* and performance indicators (see [11] for other formulations of Fitts' law). In the *high precision* condition, there were 8 targets, i.e., 8 intervals of diameter 12.5% each.

There were two modalities for the task. In the *impulsion modality*, the target was reached by means of a fast muscular contraction and the participant relaxed immediately. This corresponds to movements in which the effector leaves the target immediately after reaching it, such as in rapid alternating movements, e.g., [10]. In the *sustained modality*, the participant maintained the contraction after reaching the target so that the amplitude remained steady for one consecutive second. If stabilization was impossible within 10 s after the beginning of a trial, this trial was counted as a failure. This second task is analogue to discrete movements in which the effector remains in the target after the end of the movement, e.g., [35]. However, whereas it is generally easy to stabilize a limb or an effector in a target, this is not the case with EMG amplitude and failure could occur even though the target was reached if the participant failed to stabilize the myoelectric amplitude.

### Procedure

Participants were instructed to relax before each trial so that the EMG amplitude was below level *L* (i.e., the feedback bar was not visible). One second later, a blue rectangle appeared around the target to reach. In the impulsion modality, participants were instructed to reach the target as quickly as possible and to relax immediately. At the end of the trial, feedback was provided by having the rectangle turn green on a successful trial or orange if the trial was failed (Fig. 1c).

In the sustained modality, participants were instructed to reach the target as quickly as possible and to maintain the feedback bar within the target for at least one second. A horizontal progress bar indicated the time elapsed when on the target (Fig. 1c). When the amplitude moved out of the target, the progress bar was reinitialized. The trial ended successfully when the progress bar was completed (after 1 s). If stabilization was impossible within 10 s after the presentation of the target, the trial was counted as a failure.

Participants executed the task in four conditions, in the following order: sustained low precision, sustained high precision, impulsion low precision, and impulsion high precision. For each condition, participants performed four blocks of 32 trials. In each block, the targets appeared at random positions with a uniform probability. A two-minute pause was taken between each block in order to rest the muscles. A recalibration of the gain was performed between each block in case the range of effective myoelectric amplitudes had changed due to fatigue, perspiration and/or small displacements of the electrode montage. Recalibration was also possible within a block, e.g., when the electrode montage was accidentally displaced. Recalibration was marginal with young participants (11 times in 304 blocks), but relatively frequent with aged participants (99 times in 368 blocks). The total duration of the test was between 1.5 and 2 hours.

### Data capture and analysis

The independent variables were the distance of the target *A* (for *target amplitude*) and the diameter of the target *W* (for *target width*), determined by the condition of precision. In the impulsion modality, the performance indicators were the rate of failure *F* and the reach time *R*. The reach time was the time between the raise of the amplitude (first instant after the presentation of the target at which the smoothed signal was above the minimum *L*) and the first peak of amplitude (first instant after the raise of amplitude that was followed by two consecutive decreases of the smoothed signal). The trial was successful when the amplitude of the first peak was within the target. Note that the impulsion modality does not allow to correct overshoots and undershoots, because the amplitude is captured at the first decrease of the signal (in movement, this would be similar to the first turn back of the trajectory). This limits significantly the possibility of corrective commands. However, corrections are still possible, for instance under the form of variations in the rate of raise. This had been observed in pilot experiments but this type of event was not processed in the current data analyses.

In the sustained modality, the *reach time (R)* was determined as the time between the raise of amplitude and the moment when the amplitude entered within the target for the first time. We did not consider the first peak of amplitude as the end of the reach phase, because the objective of the participant was to stabilize the amplitude within the target and the first peak could be arbitrarily delayed (e.g., the amplitude increases slowly during the stabilization period). In order to examine the stabilization phase, we used a second dependent variable, the *complete stabilization time* (*S*), i.e., the time between the presentation of the target and the end of the first stabilization period, when the amplitude had remained within the target for one second. The difference between the complete stabilization time and the reach time is an estimate of the duration of the stabilization phase. We did not use the difference ET-RT as dependent variable because there may be trade-offs between the reach time and the stabilization, i.e., a faster stabilization (small ET-RT) may result from a smooth reach phase (large RT). In order to determine the rate of failure F, the trials were considered successful when the complete stabilization time *S* was lower than the 10s limit. Note that in the analyses, *S* was only considered in successful trials because in the event of a failure, S is equal to the 10 s time limit, which has no behavioral meaning.

The following analyses were conducted. First, we examined how age affects performance according to the task. The task is here defined by the modality and the precision demand. We performed analyses of variance (ANOVAs) on the dependent variables, with the factors of Age (between-subject) and Precision (within-subject). The two modalities were examined separately. Direct contrasts across modalities would be pointless since they correspond to different tasks that have markedly different execution times and levels of difficulty (as shown by the results of [9]).

Second, we examined the possible effects of age on the speed-accuracy trade-offs. ANOVAs with factors of Age (inter-subject) and Amplitude (intra-subject) were conducted separately for each condition of precision. We did not analyze the two conditions of precision together because Precision and Amplitude are interdependent: the number of modalities of the amplitude factor (4 vs. 8) is determined by Precision. If classical laws like Fitts' law are verified, performance should vary monotonically with amplitude, therefore if age affects the speed-accuracy trade-offs, then Age should interact significantly with Amplitude.

Finally, we performed correlational analyses in cases where a significant effect of Amplitude was observed (otherwise the correlation coefficient of a linear regression would be meaningless). Specifically, we determined the correlation coefficients and the coefficient of the regression line between performance indicators and Fitts' index of difficulty *Id = log_2_(2A/W)* (*A:* amplitude of the center of the target, *W:* width of the target), and we compared these coefficients across age groups (aged vs. young). Note that the diagrams of the dependent variables as a function of amplitude and precision are presented in all cases in order to provide a qualitative insight on the actual effect of amplitude, even if the statistical effect is not significant (for instance in the case of bow-shaped curves).

## Results

### Effects of age and precision demand on performance

**Figure 2:**
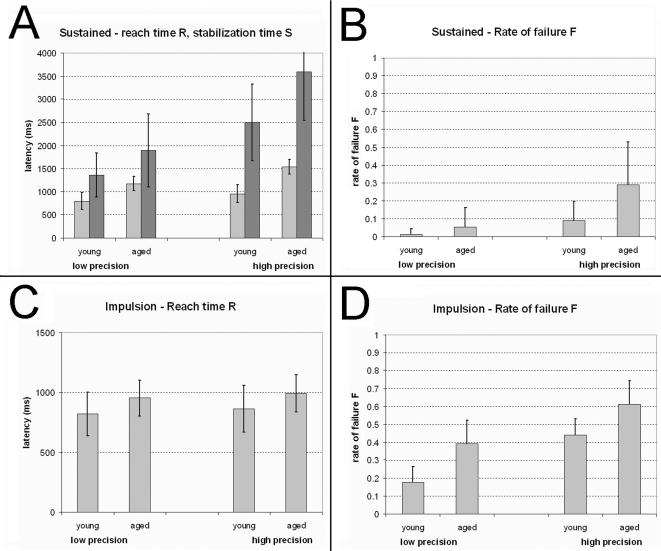
Performance indicators for young and aged participants. *A*: sustained modality, reach time R (*light grey*) and complete stabilization time S (*dark grey*). *B*: sustained modality, rate of failure; *C*: impulsion modality, reach time R. Note that the vertical axes of the graphs in *A* and *C* differ given the different ranges of response latencies in the sustained and impulsion modalities. *D*: impulsion modality, rate of failure.

There was a significant age-related decrease in performance for all the indicators in both the sustained and impulsion modalities, as shown in Fig. 2. The outcome of the ANOVAS conducted on these data is presented in Table 1.

**Table 1:** Effects of Age and Precision on performance.

Modality	Variable	Age	Precision	Age × Precision
sustained	reach time R	F(1,40) = 24.7 p<.001, Δ = 43%	F(1,40) = 45.2 p<.001, Δ = 47%	F(1,40) = 7.0 p<.05
	complete stabilization time S	F(1,40) = 14.8 p<.001, Δ = 35%	F(1,40) = 94.3 p<.001, Δ = 121%	F(1,40) = 3.7 N.S.
	rate of failure F	F(1,40) = 11.1 p<.005, Δ = 107%	F(1,40) = 30.8 p<.001, Δ = 181%	F(1,40) = 7.8 p<.01
impulsion	reach time R	F(1,40) = 7.0 p<.05, Δ = 14%	F(1,40) = 3.9 N.S.	F(1,40) = 0.0 N.S.
	rate of failure F	F(1,40) = 646.9 p<.001, Δ = 48%	F(1,40) = 308.6 p<.001, Δ = 119%	F(1,40) = 2.5 N.S.

Significance threshold is p = .05. Δ = amplitude of effect determined from estimates of marginal means, expressed in percentage of overall Mean.

Age had a significant effect on all the performance indicators (Table 1; Fig. 2), with poorer performance (i.e. greater latencies or failure rates) in the elderly than the young participants. The effects of greatest magnitude pertain to accuracy: the rate of failure is 48% higher for older than younger participants in the impulsion modality, and 107% higher in the sustained modality (see Table 1 for details). The magnitude of the age effect on execution times is somewhat smaller (reach time, impulsion modality 14%, reach time sustained modality 43%, complete stabilization time 35%).

The effect of Precision is also significant on all the indicators except for reach time in the impulsion modality (Table 1; Fig. 2). This exception is compatible with the results reported in [9], which showed that reach time is relatively constant across precision conditions in the impulsion modality, but that accuracy (rate of failure) varies markedly according to this factor, i.e., participants do not trade speed for accuracy. Note that the magnitude of the effect of precision on the performance indicators (except for reach time in the Impulsion modality, for which precision has no effect) is even greater than that of age (Table 1).

The interactions between Age and Precision are only significant in the sustained modality for reach time and the rate of failure. For both indicators, the interaction indicates a magnification of the age effect in the high precision condition compared to low precision (Fig. 2a and 2b). It is worth emphasizing the importance of the interactions between Age and Precision for the goals of the present research: significant interactions mean that Precision affects the performance of young and aged differently. If, in the same conditions, Amplitude were to affect differently the performance of young and aged participants, we would get strong support for the hypothesis that speed-accuracy trade-offs are affected by age.

### Effects of age and amplitude on performance

**Figure 3:**
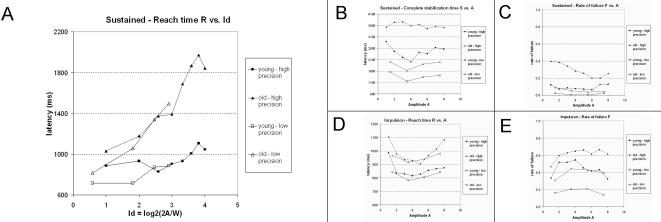
Performance indicators as functions of Amplitude and Precision. Each dot represents the average performance of one group (young, aged) for a combination of amplitude and precision. Each graph contains four curves, one per combination of Age and Precision. *A*: reach time R in the sustained modality. *B*: complete stabilization time S in the sustained modality. *C*: rate of failure F in the sustained modality. *D*: reach time R in the impulsion modality. *E*: rate of failure in the impulsion modality. Note that the horizontal scale for the reach time in the sustained modality (*A*) is Fitts' index of difficulty Id = log_2_(2A/W). This depicts how the curves in high and low precision can be fitted by a regression line. For the remainder (*B, C, D, E*), the horizontal scale is the amplitude.

The joint effects of Age and Amplitude on all dependent variables in the sustained and the impulsion modalities are shown in Fig. 3. The outcome of the ANOVAS conducted on these data is presented in Table 2.

**Table 2:** Effects of Age and Amplitude on performance, for each condition of precision.

Modality	Variable and precision	Age	Amplitude	Age × Amplitude
sustained	reach time R low precision	F(1,40) = 12.7 p<0.05, Δ = 39%	F(3,120) = 31.3 p<0.001, Δ = 44%	F(3,120) = 9.6 p<.005
	reach time R high precision	F(1,40) = 32.0 p<001, Δ = 47%	F(7,280) = 22.6 p<0.001, Δ = 46%	F(7,280) = 9.9 p<.001
	complete stabilization time S low precision	F(1,40) = 259.9 p<.001, Δ = 32%	F(3,120) = 5.8 p<.005, Δ = 24%	F = 0.41 N.S.
	complete stabilization time S high precision	F(1,40) = 458.5 p<.001, Δ = 38%	F(7,280) = 1.2 N.S.	F(7,280) = 1.9 N.S.
	rate of failure F low precision	F(1,40) = 6.6 N.S.	F(3,120) = 3.6 N.S.	F = 1.5 N.S.
	rate of failure F high precision	F(1,40) = 11.2 p<.005, Δ = 105%	F(7,280) = 3.0 N.S.	F(7,280) = 3.1 N.S.
impulsion	reach time R low precision	F(1,40) = 6.6 N.S.	F(3,120) = 3.7 N.S.	F = 0.6 N.S.
	reach time R high precision	F(1,40) = 5.6 N.S.	F(7,280) = 9.2 p<.001, Δ = 19%	F(7,280) = 1.0 N.S.
	rate of failure F low precision	F(1,40) = 38.4 p<.001, Δ = 74%	F(3,120) = 5.4 p<.005, Δ = 31%	F = 1.6 N.S.
	rate of failure F high precision	F(1,40) = 24.1 p<.001, Δ = 32%	F(7,280) = 7.3 p<.001, Δ = 37%	F(7,280) = 2.5 N.S.

Significance threshold is p = .05. The Greenhouse-Geisser corrective coefficient has been applied to the values of p because the distributions are not spherical, according to the Mauchly test. Δ = magnitude of effect determined as the maximal differences between marginal means expressed in percentage of overall Mean (unlike average difference, maximal difference allows comparing magnitude for variables that have different numbers of modalities, 2, 4 and 8).

The only dependent variable that showed a significant interaction between Age and Amplitude is the reach time (R) in the sustained modality in both the low and high precision conditions (Table 2; Fig. 3a). In addition to this interaction, this dependent variable also showed significant main effects of Age and Amplitude in both conditions of precision. It is noteworthy that this was the only dependent variable that increased monotonically with amplitude in agreement with the predictions of Fitts' Law (Fig. 3a). This finding replicates a key observation reported in [9]. The correlational analysis confirmed this result: The Pearson correlation coefficient between R and Fitts' index of difficulty *Id* was r = 0.79 for the aged participants (p<.01) and r = 0.96 (p<.01) for the young. The interaction between Age and Amplitude is reflected by a clear difference in the slope of the regression line of R as a function of Id: *R = 86.1 Id+678.6* for young participants vs. *R = 327.4 Id+572.0* for aged participants. Thus, the slope was about four times steeper for aged than for young participants, which indicates that the increased latency of the aged participants is magnified at greater amplitudes

For the remainder of the dependent variables, no interaction of Age × Amplitude was significant, thereby indicating no Age effect on the trade-offs (i.e. on the variation of performance indicators as function of target amplitude). Amplitude had significant effects on some variables (sustained modality: complete stabilization time in the low precision condition; impulsion modality: reach time in the high precision condition, and rate of failure in the high and low precision conditions; see Table 2), but no dependent variable increased monotonically as a function of amplitude. Instead, the response patterns were either flat, bow-shaped or even decreasing (Fig. 3).

### Individual performances of young vs. aged participants

Although the foregoing analyses provide clear evidence for age-related differences in myocontrol, they say little of the individual performance of aged participants. To be more specific, it would be of interest to determine whether all aged participants perform more poorly than young people or whether there is a subset of good aged performers, in agreement with the literature on successful aging [36], [37]. This question can at least be qualitatively answered by representing each participant through his performance indicators, e.g. by a dot in a plane of Latency versus Rate of Failure. With this planar representation, we obtain a visual representation of the global speed-accuracy tradeoff of each participant, whether it is a deliberate strategy (e.g., an individual may globally sacrifice speed for accuracy, or vice-versa) or the result of limited capacities (e.g. an individual's performance is poor overall).

**Figure 4:**
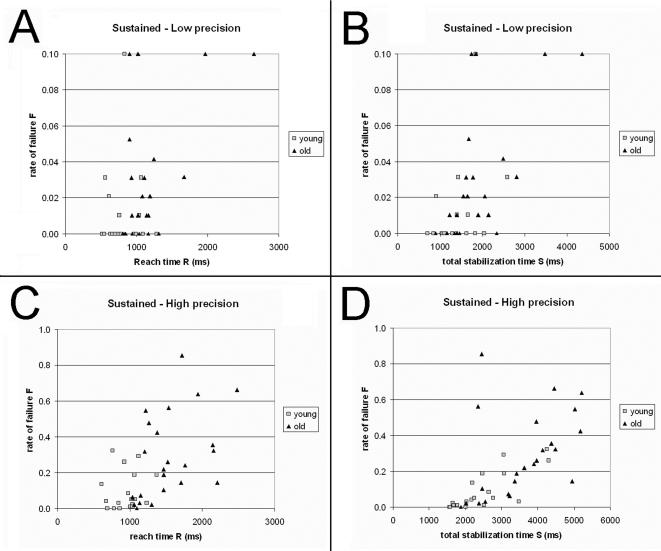
Scatter plot of performance for the sustained modality. Each dot represents the performance of one participant. *Black triangles*: old participants. *Grey squares*: young participants. The triangles that are in the middle of the group of squares represent old participants whose performance is indistinguishable from that of young people. *Vertical*: rate of failure F. *Horizontal*: time (ms). *A*: low precision, reach time R vs. F. *B*: low precision, total stabilization time S vs. F. *C*: high precision, reach time R vs. F. D: high precision, total stabilization time S vs. F. Note that the vertical axes of the graphs differ given the different ranges of rate of failure in low and high precision conditions. In addition, in *A*, *B*, the rate of failure is bounded to 0.1 for clarity.

This type of analysis was conducted on the data obtained in the sustained modality. It can be observed in Fig. 4 that there is a subset of aged participants whose performances are well within the range shown by young participants. In order to avoid any misinterpretation, it is worth emphasizing that the dots of Fig. 4 only provide information on the overall performance of each participant, but they say nothing about the underlying speed-accuracy trade-offs (the dots are averages in which the data from all the combinations of amplitude and precision are averaged).

## Discussion

### Some aspects of myocontrol are invariant with age

In many respects, the patterns of performance of young and old participants are similar. The impulsion modality, which assesses the ability to quickly raise the myolectric amplitude to a determined level, is a difficult task in which participants both young and old seem unable (or unwilling) to trade speed for accuracy. When the precision demand is increased, speed is only slightly affected, but accuracy drops dramatically. Also, the patterns of performance as a function of target amplitude are bow-shaped, in clear contradiction of Fitts' law. It is unlikely that this result is due to boundary effects, because undershoots and overshoots are equally possible. The bow shape therefore suggests that reaching extreme amplitudes is more easily done than reaching intermediate amplitudes for all participants, both young and old.

In the sustained modality, which assesses the ability to reach a determined level *and* stabilize the myoelectric amplitude, the complete execution time does not follow the predictions of Fitts' law by any means (whereas reach time does). This is compatible with the view that stabilizing the EMG amplitude in itself is a task, which may require additional time that cannot be predicted by classical movement laws. This is of importance when myolectric signals are used as entry for adaptive interfaces (e.g. visual keyboards), because one of the classical modalities of click requires stabilization (the so-called *dwelling time*: the entry must remain steady on some component, then a click occurs).

Finally, the fact that the reach time in the sustained modality is compatible with the predictions of Fitts' law, whereas it is not in the impulsion modality, supports the view that the strategy for reaching a determined myoelectric amplitude is adapted to the final state: i.e. stabilization vs. immediate return to rest level. This result was also found in [9] when the electrode was placed on the forehead instead of the hand. The reach time was calculated differently in the two modalities (sustained: time of entry in the target; impulsion: time of first peak). However, simple arithmetic considerations discard this difference as a possible cause for the result: RT was smaller in the impulsion than the sustained modality (young: 840ms vs. 875ms; aged: 970ms vs. 1358ms), and taking the time of entry instead of the time of the first peak would cause an even greater difference. Alternatively, the possible role of psychological factors like the anticipation of failure in the impulsion modality cannot be discarded. Regardless of whether the difference is caused by the final state and/or the anticipation of failure, we can conclusively assume that participants used adaptive, task-dependent strategies for reaching the intended myoelectric amplitude.

### Performance in myocontrol decreases with age

Beyond the similarities between young and aged participants, the results indicate that the performance of the aged participants as a group is poorer than that of the young participants. In both response modalities, the greatest effect of Age was on accuracy (age increased the rate of failure by 50% in the impulsion modality and by 107% in the sustained modality). However, this has to be mitigated: in the sustained modality, low precision condition, it is so easy to succeed that Age had no significant effect on the rate of failure. The latencies also increased as a function of Age (reach time, impulsion modality: 14%, reach time, sustained modality: 43%, complete stabilization time: 35%). In summary, as in motor control, the performances in myocontrol seem significantly affected by age. This has important consequences for the usability of adaptive technology using myoelectric signals: the signal processing parameters should be adjustable in order to handle the general decrease of performance of aged users.

A closer examination of the outcome of the ANOVAs (Tables 1 and 2) brings additional insights on the effects of age on accuracy. In the sustained modality, the effect of Age on the rate of failure is significant only in the high precision condition (effect magnitude of 105%). This may be interpreted in either of two ways. The absence of a significant effect in the low precision condition may be due to a floor effect, i.e., the task was so easy that all participants, young and aged are successful. Alternatively, the importance of the effect in the high precision condition may be interpreted as signaling a fragility of myocontrol in aged participants, i.e., performance is markedly degraded as the level of difficulty is increased. The latter view is supported by the results in the impulsion modality. In the high precision condition, the rate of failure of aged participants is nearly catastrophic (above 60%) and certainly high enough to make this response modality totally impractical in applied settings. Whether the fragility of performance is attributable to a lower capacity of the neuromuscular system or of the proprioceptive system, to different mechanisms of control (covert) or different control strategies (overt), or to a combination of these factors remains an open issue.

### Speed-accuracy tradeoffs in myocontrol, when they exist, are affected by age

The only task that allowed for clear speed-accuracy tradeoffs in this experiment was that of reaching a determined myoelectric amplitude with the goal of stabilizing the signal at this level–i.e. *R* in the sustained modality. In this task, the variation of latency as a function of amplitude and precision is compatible with the predictions of Fitts' law. However, the slope of the regression line of R as a function of Fitts' index of difficulty *Id* is quite different for young and aged participants. The performance of aged participants is markedly more degraded as difficulty (as represented by Id) increases than that of young participants. The slope of the regression line has been interpreted by Fitts [10] as an information transmission coefficient, but in behavioral terms, it means that the speed-accuracy trade-off is sensitive to age: in myocontrol, aged persons have to sacrifice more speed than young persons for additional accuracy. This is compatible with the view proposed above that myocontrol in aged persons is more fragile than in young participants, i.e., the level of performance is markedly more degraded as the difficulty of the task is increased.

### Some aged participants perform as well as young people

The scatter plots of Fig. 4 depict a varied panorama. In these plots, each dot represents an individual, and the coordinates represent the overall individual speed-accuracy tradeoff. Aged participants appear to be more scattered than young persons, but the striking point is that some aged participants perform well within the range shown by young persons. It is true, however, that finer differences may exist in terms of speed-accuracy tradeoffs (because the performances that are depicted in Fig. 4 are an average across all the combinations of amplitude and precision). Also, an open question is whether the tradeoffs are deliberate, i.e., the individual chooses to sacrifice speed for accuracy or vice-versa, or this is imposed by limited capacities, that is the individual is globally slow and/or inaccurate. In any case, this result is only qualitative: the present study was not designed to quantify individual differences. However, this supports the view that the myocontrol performance of some elderly persons is relatively unaffected relative to younger individuals, just as in cognitive, memory, or motor tasks [36], [37]. This finding may be of importance for the design of adaptive technologies using myoelectric signals: they support the view that the systems should be flexible enough to handle the diversity- in addition to the age- of the users.
